# Insights into aging mechanisms from comparative genomics in orange and silver roughies

**DOI:** 10.1038/s41598-024-70642-w

**Published:** 2024-08-26

**Authors:** Dido Carrero, Maria Pascual-Torner, Diana Álvarez-Puente, Víctor Quesada, Claudia García-Gómez, Carlos López-Otín

**Affiliations:** 1 Departamento de Bioquímica y Biología Molecular, Instituto Universitario de Oncología, Ciberonc, Universidad de Oviedo, Oviedo, Spain; 2 Observatorio Marino de Asturias, Departamento de Biología de Organismos y Sistemas, Universidad de Oviedo, Oviedo, Spain

**Keywords:** Comparative genomics, Marine biology, Ageing

## Abstract

The demersal fish orange roughy (*Hoplostethus atlanticus*) can live for up to 250 years, twenty times more than its congener silver roughy (*Hoplostethus mediterraneus*). Studies of *Hoplostethus* have focused mainly on its ecology and conservation due to its vulnerability to commercial fishing. In this work, we present the de novo genomes of orange and silver roughies and explore the genomic mechanisms that could contribute to such differential longevities. Using comparative genomics on a list of more than 400 genes, we identified gene candidates with differential residue changes in *Hoplostethus* that are related to genomic instability, disabled macroautophagy and intercellular communication. We hypothesized that these mechanisms could have been selected as adaptations to the deep environment and, as an epiphenomenon of these mechanisms, may have contributed to an extension of the lifespan of *H. atlanticus*.

Ageing involves molecular processes that are conserved throughout evolution. According to recent classifications, aging features can be grouped into twelve hallmarks. These hallmarks can be (1) related to primary causes of cellular damage (genome instability, telomere attrition, epigenetic alterations, loss of proteostasis and disabled macroautophagy); (2) involved in a compensatory or antagonistic response to this damage (deregulated nutrient-sensing, mitochondrial dysfunction and cellular senescence); or (3) represent an integrative consequence of the other two categories (stem cell exhaustion, altered intercellular communication, chronic inflammation and dysbiosis)^1^. Multispecies comparative genomics in different animal taxa has revealed that genomic alterations affecting one or several hallmarks are candidates that contribute to differential interspecific longevity^2–5^.

Fishes are a vertebrate taxon with a wide range of lifespans, varying from weeks in pygmy gobies^6^ to approximately 400 years in Greenland sharks^7^. Large differences even occur among species within the same genus. For example, the longevity of rockfish ranges from 11 years in *Sebastes minor* to more than 200 years in *Sebastes aleutianus*. Similarly, species of the genus *Hoplostethus* have lifespans that vary between 11 years (*Hoplostethus mediterraneus*) and 250 years (*Hoplostethus atlanticus*)^8–10^. In terms of the habitat range of these two *Hoplostethus* species, both species are demersal: *H. atlanticus* lives on the slope of the continental shelf and on seamounts from 700 to 1300 m depth^11^, and *H. mediterraneus* is mostly distributed in habitats from 500 to 750 m depth^12^ (Fig. 1). *Hoplostethus mediterraneus* is often caught as a bycatch species via bottom trawling, while *H. atlanticus* has been of commercial interest for decades^12^. Regarding their biology, *H. mediterraneus* has a maximum length of 198 mm and reaches maturity at sizes greater than 115 mm, which corresponds to an age of approximately 2 years^13^. In contrast, *H. atlanticus* can measure up to 500 mm^14^ and grows slowly until it reaches sexual maturity at approximately 20–30 years, which makes this species highly vulnerable to overfishing^11^. For this reason, research on *Hoplostethus*, especially *H. atlanticus*, has focused exclusively on ecology and conservation, as well as on reporting the state of its populations around the world^9^, whereas less attention has been given to exploring the extreme differences in their lifespan.

**Fig. 1:**
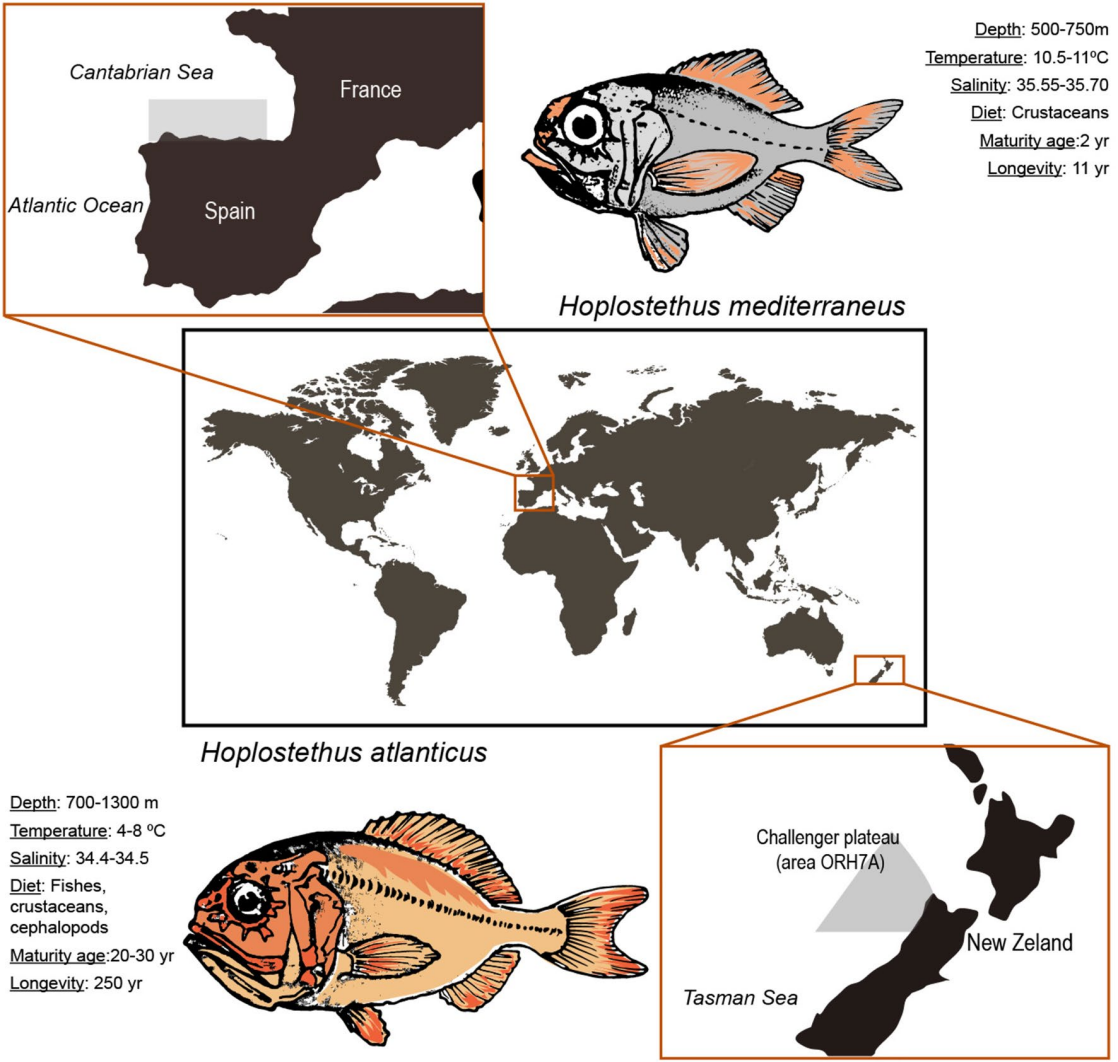
Locations from where samples of (top) *H. mediterraneus* and (bottom) *H. atlanticus* were collected. Depth ranges^11,12^, temperature and salinity of *H. mediterraneus* depth range^81^, diets^45,46^, age at maturity^12,13^ and longevity^8–10^ were extracted from the literature, whereas the temperature and salinity ranges for *H. atlanticus* were extracted by plotting depth profiles (Supplementary information Fig. S2) via Argo buoy data^82^. The drawing of the world map and the specific geographic areas were designed using an image from Freepik (https://www.freepik.es/).

In this work, we sequenced and assembled the genomes of *H. atlanticus* and *H. mediterraneus *de novo, performed manually supervised annotation of a set of more than 400 genes related to aging, and used comparative genomics to select gene amplifications and residue changes specific to either of these species that could contribute to the 20-fold difference in longevity of *Hoplostethus sp.*

## Results and discussion

### Genome assembly

The assembly of *H. atlanticus* has a total size of 634 Megabases (Mb), with 1436 contigs with an N50 of 3290 kb. The assembly of *Hoplostethus mediterraneus* has 552 Mb with 657 contigs and an N50 of 3054 kb. Despite the smaller size and lower number of contigs of the *H. mediterraneus* assembly, both have a high degree of completeness, with more than 91% Benchmarking Universal Single-Copy Ortholog (BUSCO)^15^ complete genes and a similar GC content of approximately 43% (Table 1). Automatic annotation led to 74,284 and 73,650 predicted protein-coding genes in *H. mediterraneus* and *H. atlanticus*, respectively. The total number of repetitive elements (Supplementary Table S2) spanned 17.77% of the *H. atlanticus* genome and 13.85% of the *H. mediterraneus* genome, similar to values reported in other fish genomes, such as *Nothobranchius furzeri* (15.86%)^16^. Compared with *H. mediterraneus*, *Hoplostethus atlanticus* presented higher percentages of most repetitive element types, which could be explained by its greater genome size^17^ but slightly lower simple and low-complexity repeats. Simple repeats were the most abundant elements in both *Hoplostethus species*, and retrotransposons were infrequent compared with those in other species, such as the short-lived *N. furzeri*^16^.

**Table 1:** Results of the assembly of both *Hoplostethus* species.

	*H. mediterraneus*	*H. atlanticus*
Estimated size	552,071,163	634,153,829
Heterozygosity	0789	0757
Contigs	657	1436
Contig-N50	3,053,845	3,289,651
Contig-max lentgh	19,549,029	23,036,873
Contig-mean	442,049	916,952
Mapped reads	81,030,860 (99.46%)	98,599,251 (99.63%)
Mapped reads- coverage	98.63%	97.92%
BUSCO-Complete and single-copy	92.41%	91.42%
BUSCO-Complete and duplicated	3.96%	4.29%
BUSCO-Fragmented	0.33%	0.33%
BUSCO-Missing	3.30%	3.96%
GC content	43.65%	43.34%

### Hypothesis-driven manual annotation

**Fig. 2:**
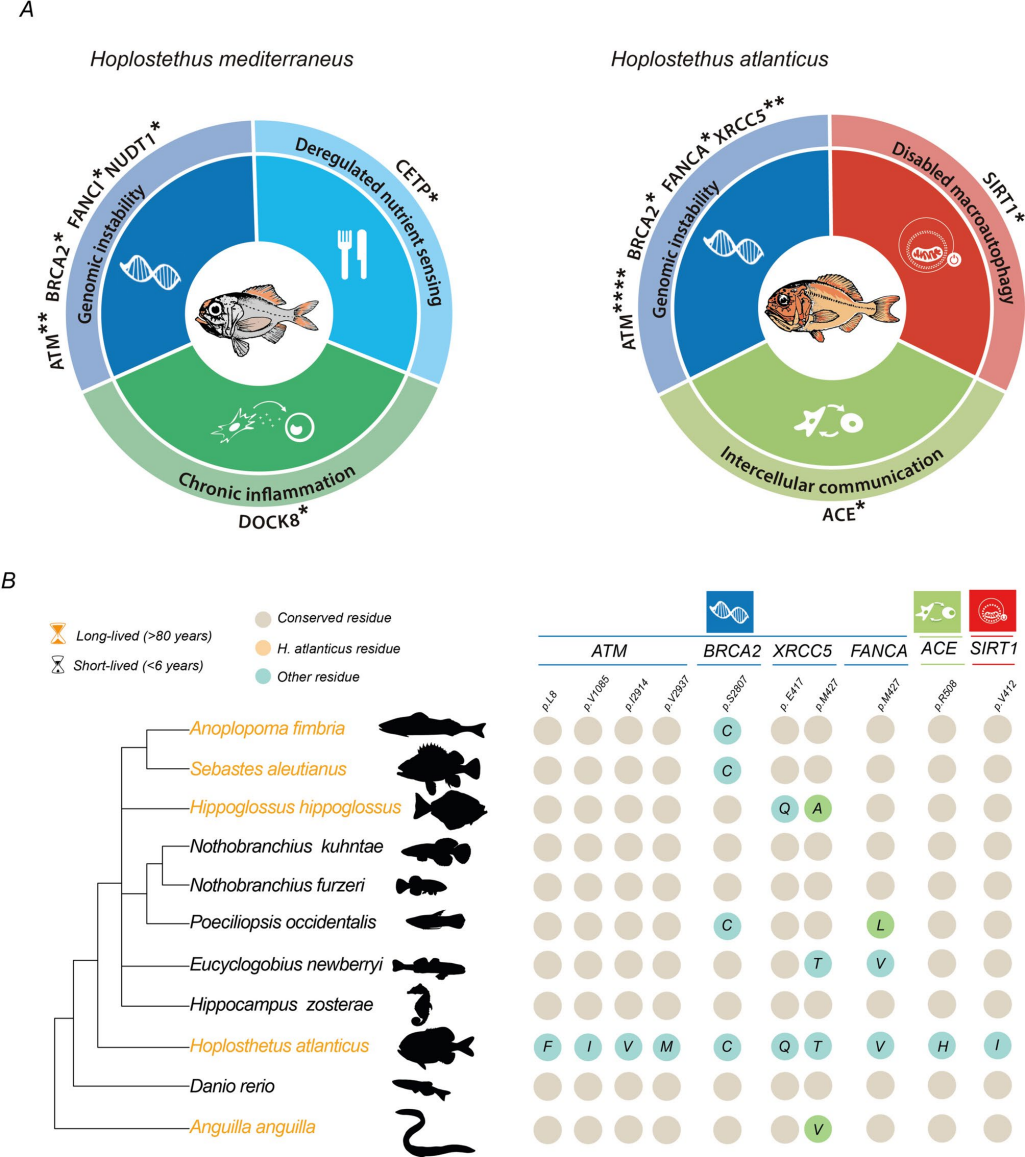
Genomic bases of longevity in *Hoplostethus*. (**A**) Candidate genes contributing to differential longevity between *H. mediterraneus* (left) and *H. atlanticus* (right), classified by the hallmarks of aging. * Refers to the number of point variants of interest found in each gene. (**B**) Residue changes are more relevant in *H. atlanticus* than in other long- and short-lived fishes.

Using manually supervised annotation, we compared the status of more than 400 genes linked to aging and longevity between *H. mediterraneus* and *H. atlanticus* (Supplementary Table S3) and identified multiple amplified genes and residue changes in comparison to the other species analysed. From these results, we selected those with differential changes between the two *Hoplostethus* species, resulting in a set of 19 sequence variants (Table 2 and Fig. 2). Notably, all changes in gene amplification and several point variants found in candidate genes were discarded after PCR validation (see Supplementary Tables S4 and S5). This underscores the importance of integrating experimental validation with bioinformatics analysis to yield more reliable and robust results.

**Table 2:** Genes of interest selected after manual annotation, together with their RNA-seq, PCR and genomic validations**,** predictions of effects in humans following the SIFT and Poly-Phen models**,** and Clinvar accession number of information related to the residue position.

*Gene*	*Variant*	*Species*	RNA-seq validation	Genomic validation	PCR validation	SIFT	PolyPhen	Clinvar accession^2^
*ACE*	R508H	*Hatl*	Yes (50%)	Yes (50%)	yes	T (0.57)	PsD (0.757)	–
*ATM*	R184Q	*Hmed*	yes	yes	yes	APF (0.00)	PbD (0.992)	VCV000481315.7
*ATM*	Q2177R	*Hmed*	yes	yes	yes	APF (0.00)	PbD (0.999)	VCV001810514.4
*ATM*	L8F	*Hatl*	yes	yes	yes	APF (0.03)	PbD (1.00)	VCV000487013.7
*ATM*	V1085I	*Hatl*	yes	yes	yes	APF (0.01)	PbD (0.995)	VCV000453455.2
*ATM*	I2914V	*Hatl*	yes	yes	yes	APF (0.03)	B (0.208)	VCV000187320.22
*ATM*	V2937M	*Hatl*	yes	yes	yes	APF (0.01)	PsD (0.929)	VCV000187681.3
*BLM*	M827V^1^	*Hatl*	yes	yes	yes	T (0.10)	PbD (1.00)	–
*BLM*	I1039V^1^	*Hatl*	yes	yes	yes	T (0.11)	B (0.004)	–
*BRCA2*	S2807R	*Hmed*	yes	yes	yes	APF (0.00)	PbD (0.982)	VCV000216033.5
*BRCA2*	S2807C	*Hatl*	yes	yes	yes	APF (0.00)	PsD (0.461)	VCV000441481.16
*CETP*	V29A	*Hmed*	no reads	yes	yes	APF (0.02)	B (0.001)	–
*DOCK8*	R398H	*Hmed*	yes	yes	yes	APF (0.00)	PbD (0.999)	VCV000235481.7
*FANCA*	M427V	*Hatl*	yes	yes	–	–	B (0.271)	VCV001023721.1
*FANCI*	S979T	*Hmed*	yes	yes	yes	–	B (0.230)	VCV000408250.1
*NUDT1*	G99S	*Hmed*	yes	yes	yes	APF (0.00)	PsD (0.772)	–
*SIRT1*	V412I	*Hatl*	yes	yes	yes	APF (0.03)	PsD (0.925)	–
*XRCC5*	E417Q	*Hatl*	yes	yes	yes	T (0.29)	PsD (0.770)	–
*XRCC5*	M427T	*Hatl*	yes	yes	yes	APF (0.00)	B (0.128)	–

B: Benign; PbD: Probably damaging; PsD: Possibly damaging; T: Tolerated; APF: Affect protein function. *Hmed: H. mediterraneus*, *Hatl: H. atlanticus.*

^1^Note that in *Drosophila melanogaster* variants are M827I and I1039A.

^2^Only one Clinvar accession number is shown, even if there was more than one for each position.

The molecular and cellular mechanisms underlying the process of aging have recently been categorized into twelve hallmarks, which are conserved throughout evolution^1^. These hallmarks are relevant because they are associated with alterations that increase over time, can be experimentally controlled in models that mimic accelerated aging, and can be used to understand how each specific hallmark contributes to the overall aging process. Finally, they permit the identification of potential targets to decelerate, cease, or reverse aging through therapeutic interventions targeting each specific hallmark^1^. The genes that we propose as candidates to play a role in the long-lived phenotype shown by *H. atlanticus* can also be grouped into the following twelve hallmarks: genomic instability, telomere attrition, epigenetic alterations, loss of proteostasis, disabled macroautophagy, deregulated nutrient sensing, mitochondrial dysfunction, cellular senescence, stem cell exhaustion, altered intercellular communication, chronic inflammation, and dysbiosis. Importantly, although we classified each candidate gene into a single hallmark for the purpose of organizing the results, some genes can be involved in more than one function or mechanism.

#### Genomic instability

The accumulation of genetic damage resulting in genomic instability can challenge tissue homeostasis and accelerate aging^18^, modulating species longevity. DNA integrity can be compromised not only by exogenous factors, including biological, physical^19^ (such as hydrostatic pressure) or chemical agents^20^ but also by endogenous factors, such as reactive oxygen species (ROS) or DNA replication errors^21^. Genomic instability can be prevented by having both efficient replicative mechanisms and DNA repair systems. Consequently, we explored several genes involved in the maintenance of DNA integrity. This analysis revealed that *H. atlanticus* presents two variants (p.E417Q and p.M427T) in the heterodimer interface of the protein XRCC5^22^. Heterodimerization with XRCC6 is crucial for NHEJ-dependent DNA repair. The residue p.M427 is conserved only in vertebrates, and changes in this position are also found in two other long-lived fishes (*Hippoglossus hippoglossus*, p.M427A; *Anguilla anguilla*, p.M427V) and in one short-lived fish (*Eucyclogobius newberryi*, p.M427T). The residue p.E417 is also conserved within fungi, and the same p.E417Q variant can also be found in the long-lived *H. hippoglossus* but is absent in all short-lived fishes analysed (Fig. 2B).

**Fig. 3:**
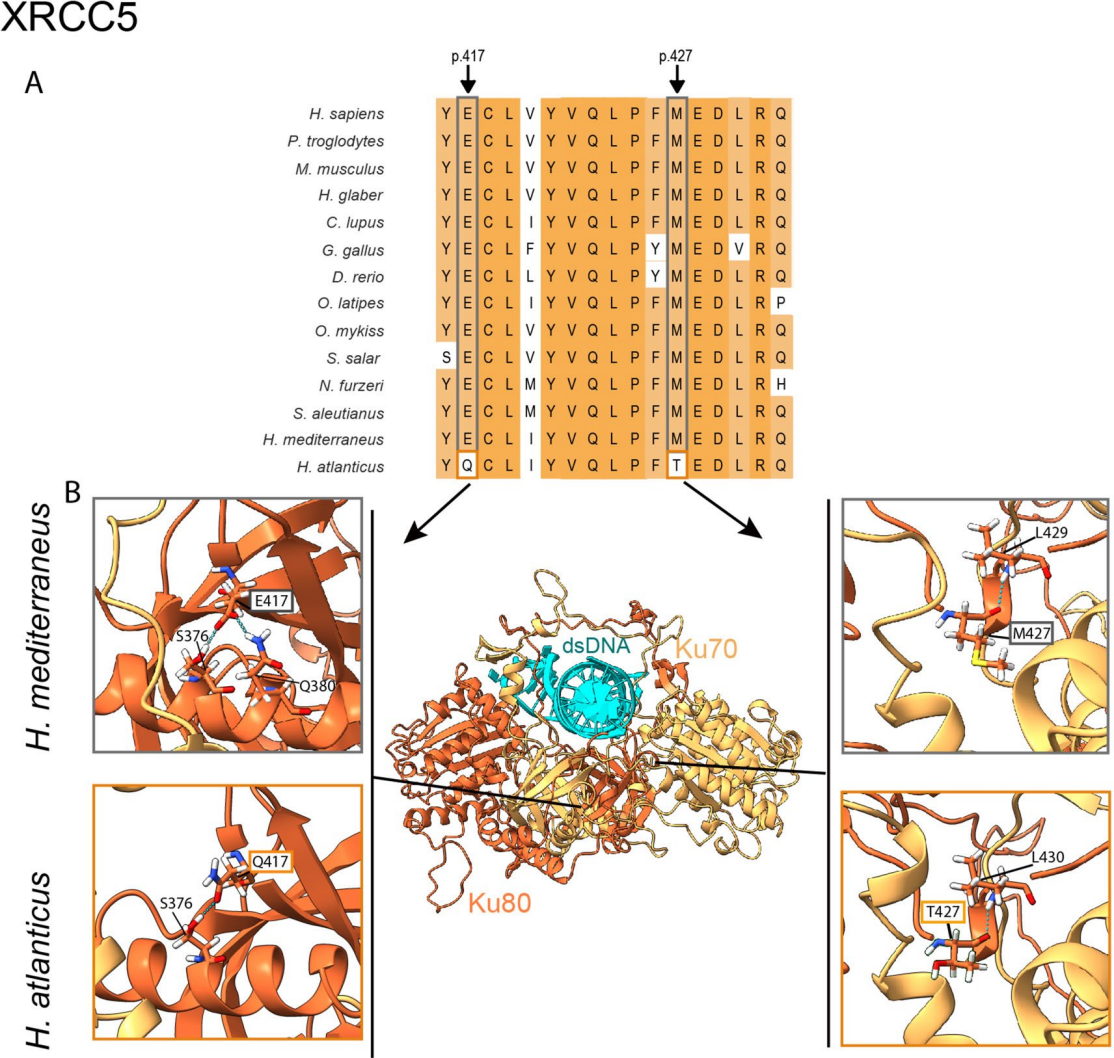
Protein sequence comparison and homology modelling of XRCC5. A. Partial amino acid sequence alignment of both *Hoplostethus* and other fish, vertebrate and invertebrate species, where the positions p.417 and p.427 are highlighted, and B. Model of the human heterodimer Ku70-Ku80 with interaction with dsDNA^83^ and the specific changes on p. 417 and p. 427 in *Hoplostethus*. The dashed blue lines indicate H-bonds.

A homology model of the XRCC5 protein suggested that the p.M427T variant reduces intramolecular hydrogen bonding (Fig. 3), which could modify flexibility in heterodimerization and therefore modulate the efficiency of XRCC5-dependent DNA repair mechanisms. Deletion of *XRCC5* results in early apoptosis of human cells^23^ and, in mice, leads to deficiencies in the immune system and early senescence^23,24^. Its heterodimer counterpart, XRCC6, is also involved in aging, since knockout of *Xrcc6* in mice results in decreased lifespan^22^, and high expression of *XRCC6* in humans leads to a longer average lifespan^25^. In fish, Ku80 has been associated with DNA repair during stages of development in zebrafish exposed to irradiation stress^26^. Additionally, recent comparative analyses of ~ 1000 mammalian species have shown that this gene likely plays a role in both longevity and social organization^27^. Hence, we propose a moderate effect for these variants, particularly for the change affecting residue p.E417, in XRCC5 function and in *Hoplostethus* lifespan.

We also found several residue variations affecting the gene encoding the serine/threonine protein kinase ATM, a protein that acts as a DNA damage sensor and is located in highly conserved positions. First, we discovered four different variants in *H. atlanticus* affecting protein function: two (p.L8F and p.V1085I) in the N-terminal region, which are predicted to be damaging in humans (Table 2), and another two (p.I2914V and p.V2937M), which belong to the kinase domain^28^, are predicted to be benign and possibly damaging in humans, respectively (Table 2). These changes were not detected in any other short- or long-lived fishes (Fig. 2B). In *H. mediterraneus*, we found two variants (p.R184Q and p.Q2177R), both of which are predicted to affect protein function and be probably damaging in humans. All the positions of the preceding point variants in ATM have been associated with cancer predisposition and/or ataxia-telangiectasia syndrome (Table 2). These findings would suggest a relevant role for both positions in ATM evolution.

The gene *BRCA2* encodes a protein that is involved in double-strand break repair and homologous recombination. We observed a change in the residues p.S2807 to Cys in *H. atlanticus* and to Arg in *H. mediterraneus*, which changed from polar to nonpolar and positively charged, respectively. Changes in this position have been associated with hereditary breast and ovarian cancers (Table 2). This highly conserved residue is in the oligonucleotide binding fold (OB1), which interacts with the exoribonuclease DSS1^29^. Cys is present not only in the long-lived *S. aleutianus* and *Anoplopoma fimbria* but also in several other fishes, such as *Poeciliopsis occidentalis*, *Xiphophorus helleri*, *Poecilia reticulata* and *Oreochromis niloticus*, which have lived for less than 10 years (Fig. 2B, Supplementary Fig. S1). *BRCA2* mutations at different positions have been associated with cancer risk in adult zebrafish tissue^30^. Additionally, we detected residue changes in other components of the Fanconi anaemia complex, including a variant in FANCI at p.R393H in *H. mediterraneus* and at p.M427V in FANCA in *H. atlanticus*, both of which are related to Fanconi anaemia (Table 2). These two variants are in highly conserved positions, since the same residue is maintained in more than 80% of the species, even though few fishes, birds and mammals, including two short-lived fishes, show changes in this position (Fig. 2B). Both variants are predicted to be benign in humans (Table 2).

Finally, the variant p.G58S, which is exclusive to *H. mediterraneus*, lies within the nudix motif of the hydrolase NUDT1^31^, which appears to be important for maintaining the stable structure of the protein^32^, and changes in other residues of the nudix motif led to loss of activity^32^. This protein is involved in preventing DNA transversions and therefore plays a relevant role in protection from oxidative stress. Additionally, NUDT1 deficiency leads to increased tumor incidence in mice^33^. The abovementioned variant is predicted to be deleterious or possibly damaging in humans (Table 2). However, its effect on protein function has yet to be studied.

#### Disabled macroautophagy

Macroautophagy is a type of autophagy that involves the sequestration of cytoplasmic material into double-membrane vesicles, called autophagosomes, which then fuse with lysosomes for the digestion of their contents. It affects all kinds of macromolecules, entire organelles, and even invading pathogens^34^, and strong evidence suggests that autophagy is a relevant mechanism that regulates aging^1^.

**Fig. 4:**
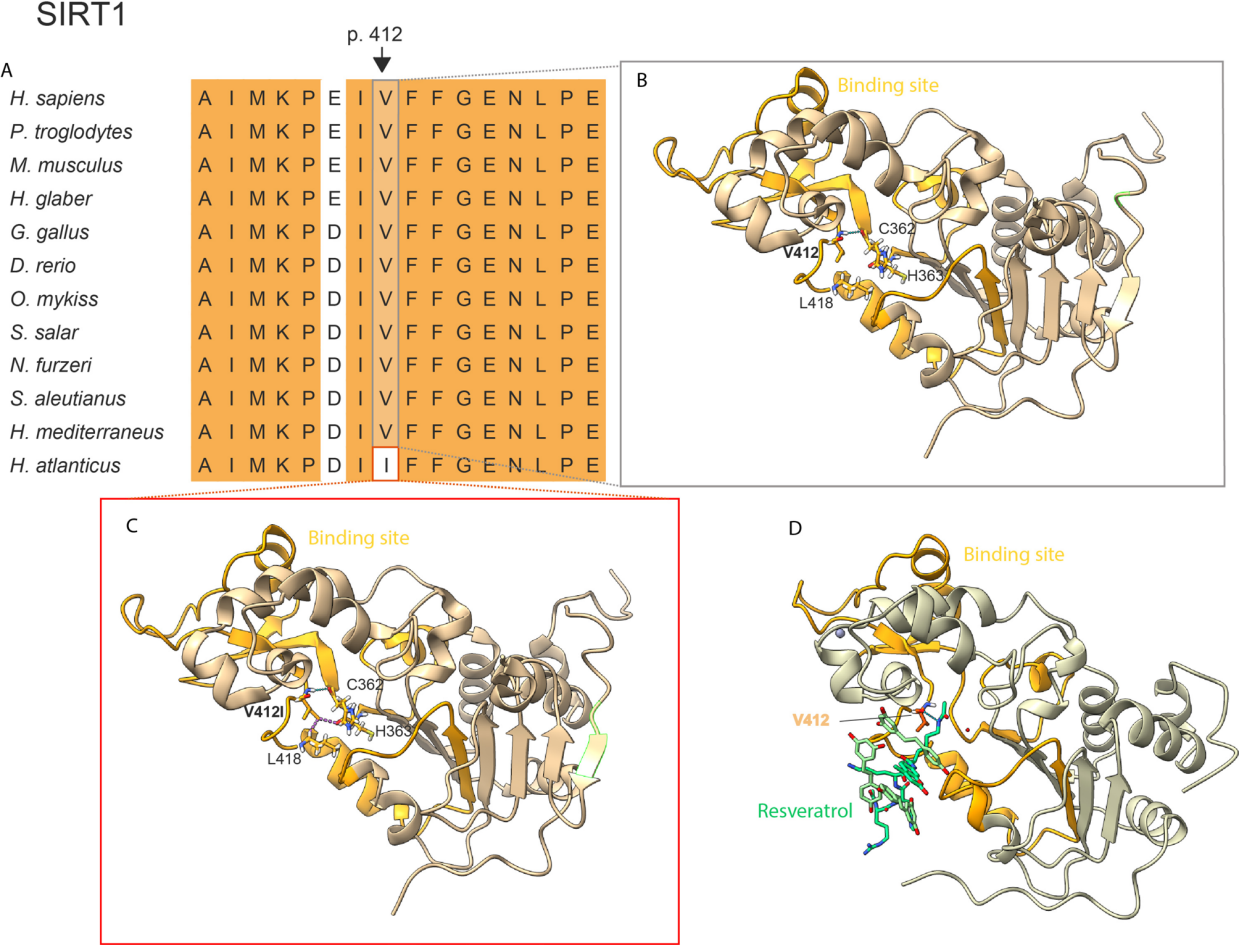
Protein sequence comparison and homology modelling of SIRT1. (**A**) Partial amino acid sequence alignment of *Hoplostethus* and other fish, vertebrate and invertebrate species; position p.412 is highlighted. This position is also represented in a protein model of SIRT1 from (**B**) *H. mediterraneus*; (**C**) *H. atlanticus;* and (**D**) a human model with interaction with resveratrol^84^. The dashed blue lines indicate H-bonds, whereas the dashed purple line indicates steric clashes.

In this context, we also discovered the variant p.V412I in the protein SIRT1, which lies in a highly conserved residue involved in substrate binding, in H. atlanticus^35^. This change results in steric clashes with L418 and H363, which can slightly modify the folding of the substrate-binding domain and is predicted to affect protein function in humans (Fig. 4, Table 2). While macroautophagy downregulates the activity of the deacetylase SIRT1 during aging and senescence, this protein is also a negative regulator of mTOR^36^, a complex that has direct implications in aging^37^. Among other relevant functions in the cell, SIRT1 induces insulin sensitivity^38,39^ and keratinocyte differentiation^40^. In addition, SIRT1 also stimulates tissue regeneration in zebrafish via modulation of the mitochondrial unfolded protein response (UPR^mt^)^41^. The regulation of sirtuin activity through NAD + precursors has many implications for different hallmarks of aging^1^. Therefore, we hypothesize that the abovementioned variant found in SIRT1 may act through different mechanisms to promote longevity in *H. atlanticus*. Further experimental approaches may contribute to shed light on the effect of this mutation in SIRT1 protein function.

#### Deregulated nutrient sensing

The regulation of nutrient sensing involves several pathways in the cell, such as the IGF-1 signalling (ISS) pathway, which in turn modulates other pathways associated with longevity, such as the FOXO, sirtuin and mTOR pathways. High nutrient availability activates signalling pathways that promote aging, whereas nutrient scarcity has the opposite effect^42^. Moreover, integrative transcriptomic analyses of more than 40 mammalian species have recently revealed shared longevity mechanisms that act as biomarkers of longevity and ageing, including downregulation of *IGF1*^37^. With respect to nutrient sensing, we found a variant exclusive to *H. mediterraneus* in *CETP*, a gene that codes for a protein in the plasma that is involved in cholesterol and triglyceride transport^43^. This variant p.V29A is in a highly conserved position within the BPI dimerization interface^44^; however, the same residue change is present in some birds and fishes, such as *Latimeria chalumnae*, which presents a longevity of 48 years. This variant could be associated with the different diets of the two species, as *H. mediterraneus* preys mostly on crustaceans^45^, whereas *H. atlanticus* feeds on crustaceans only in the early stages and mostly on fish when adults^46^.

#### Altered intercellular communication

**Fig. 5:**
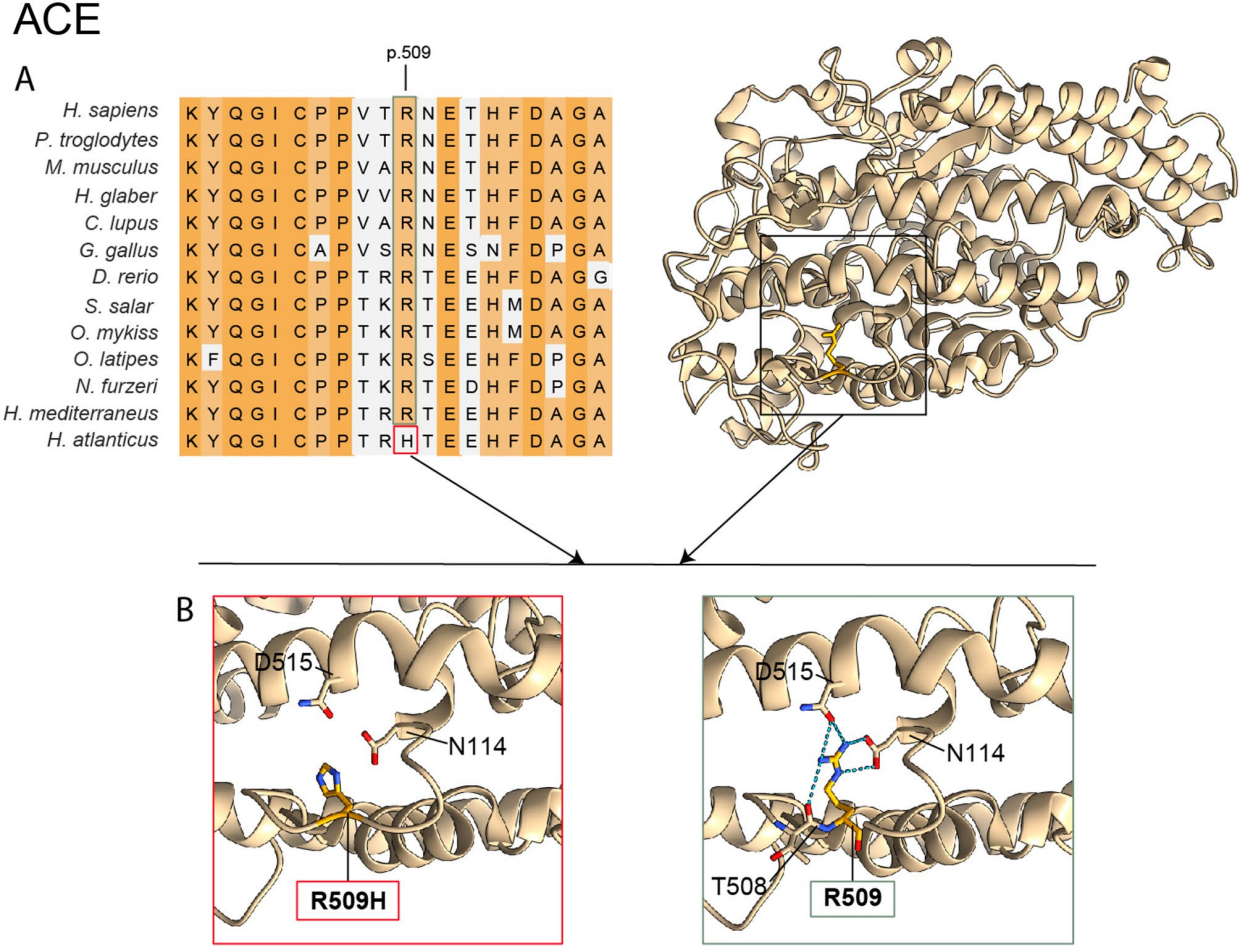
Protein sequence comparison and homology modelling of ACE. (**A**) Partial amino acid sequence alignment of *Hoplostethus* and other fish, vertebrate and invertebrate species, with the position p.509 highlighted. (**B**) Details of the amino acid interactions at this position are shown for *H. atlanticus* and *H. mediterraneus* in the protein human model^85^. The dashed blue lines indicate H-bonds.

Alterations in intercellular communication, including endocrine, neuroendocrine or neuronal pathways, can also promote aging^1^. In this context, we found a residue change (p.R509H) exclusive to *H. atlanticus* in the ACE gene, which codes for a protein that catalyzes the conversion of angiotensin I into angiotensin II, modulating its vasoconstrictor activity and osmoregulation in fish^47^. This variant has been associated with autosomal recessive renal tubular dysgenesis (RTD), a disease with symptoms of arterial hypotension, probably by affecting the renin–angiotensin system (RAS)^48^. p.R509 is a highly conserved position, and the change of Arg to His would modify H-bonds with p.D515 and p.N114 (Fig. 5, Table 2). ACE inhibition reduces angiotensin II production, which reduces arterial pressure^49^ and impairs mitochondrial dysfunction^48^, contributing to increased longevity. Moreover, high levels of angiotensin II increase blood pressure through the release of catecholamines^50^ and affect renal function by reducing parameters such as the glomerular filtration rate or urine flow in bony fishes^51^. Thus, we hypothesize that this residue change in ACE could modulate the RAS system and help control blood pressure, the antioxidant environment and osmoregulation.

#### Chronic inflammation

Inflammation is known to be closely related to aging, a trait known as “inflammaging”^5^. Given that inflammation and circulating concentrations of inflammatory cytokines increase with age, while the function of the immune system decreases, we explored several genes related to these two processes. We identified the p.R393H variant in the DOCK8 protein of *H. mediterraneus*, which is involved in T-cell and dendritic cell migration during immune responses. This variant is predicted to affect protein function and to be probably damaging in humans (Table 2), and it has been associated with autosomal recessive hyper-IgE syndrome (Table 2). Despite this variant being in a highly conserved position, the same residue change occurs in one of the three copies of the corresponding genes in *Salmo salar*, which suggests a case of parallel evolution^52^. In addition, we explored members of the butyrophilin gene family (*BTN* and *BTNL* genes), which are immune regulators involved in human inflammatory disease and are associated with depth components of lifespan in rockfish^3^. However, we did not find differences in the gene amplification of any specific gene within this family (Supplementary Table S6).

### Deep-sea environment and aging

Deep-sea marine environments are characterized by low temperatures, low food availability and high hydrostatic pressures, which can induce DNA damage and affect protein function^53,54^. Thus, maintenance of genomic stability could be relevant to address this source of damage. Accordingly, genes related to DNA repair (*ATM*, *BRCA2*, *FANCA*, *FANCI, NUDT1* and *XRCC5*) were relevant in both *Hoplostethus* species—with a slightly greater number of variants in *H.* atlanticus—which also reinforces the fact that this primary hallmark is a common denominator highlighted in many comparative genomic studies of aging, such as bowhead whale^55^, giant tortoise^2^, tardigrade^56^, immortal jellyfish^4^, killifish^16^ or rockfish^3^. Moreover, the upregulation of genes related to DNA repair was linked to longevity in mammals^57^. Interestingly, variants and amplifications in the heterodimer Ku70-Ku80 (encoded by *XRCC5* and *XRCC6*) have also been reported in other comparative genomic studies of long- and short-lived species, such as killifish, immortal jellyfish, and giant tortoise^4,16,22^. The importance of the variants found in XRCC5 in *H. atlanticus* is reinforced by the presence of the same amino acid change (p.E417Q) in another long-lived fish, *H. hippoglossus*, and by the finding of different changes in residue p.M427 in two other long-lived species (*H. hippoglossus* and *A. anguilla*) and in one short-lived fish (*E. newberryi*), which suggests a case of parallel evolution^52^. This result may indicate that this heterodimer could act as a node to modulate aging in both directions depending on the position of the residue, which is changed in different species^16^.

Hydrostatic pressure damages DNA, alters membrane fluidity, and affects proteins directly. Under high hydrostatic pressure, proteins may experience functional changes due to mechanisms such as decreased molar volume, hydration of uncharged regions, unfolding, and denaturation, while also dissociating multimeric proteins and affecting enzymatic efficiency. Deep-sea species have adapted to these conditions, including modifications in membrane lipids to maintain fluidity and changes in protein amino acid residues, which are sufficient to mitigate the negative effects of high pressure on protein function^58^. Additionally, many of these species produce protein-stabilizing osmolytes such as trimethylamine-N-oxide (TMAO) to create an intracellular environment that protects against protein instability^53,59^. TMAO levels increase in bony fishes and invertebrates as they inhabit greater depths^60^ and are significantly greater in *H. atlanticus* than in other fishes^61^. Since TMAO is synthesized by hepatic flavin monooxygenase (FMO) through the oxidation of trimethylamine (TMA)^62^, we manually annotated *FMO* family genes in *Hoplostethus* and other fishes and found that all of these fishes contain *FMO2* and *FMO5* without any differential gene amplification in *H. atlanticus* (Supplementary Table S7). When protein aggregates are formed, macroautophagy removes them along with other intracellular macromolecules, organelles, and pathogens^1^. In this context, we observed the abovementioned residue change in the protein SIRT1, an epigenetic regulator related to macroautophagy. SIRT1 is also involved in mitochondrial proteostasis in zebrafish through the mitochondrial unfolded protein response (UPR^mt^)^41^. Considering that loss of proteostasis is a primary hallmark of aging, this protective mechanism, which may help maintain functional proteins at pressures up to 100 atm (at 1000 m), could also contribute to increasing the longevity of *H. atlanticus*.

Fishes, as ectotherms, are especially affected by changes in water temperature, which affects their metabolic and growth rates^63^. At great depths, low temperatures can act in synergy with high pressures to reduce the fluidity of the cellular membrane and can also affect protein stability^58^. The temperature values within the depth range distribution of *H. atlanticus* on the Challenger Plateau were much lower than those of *H. mediterraneus* in the Cantabrian Sea. In contrast, salinity variation within the depth range was almost negligible for both species and presented a small difference among habitats (Fig. 1). Thus, the influences of low temperature and high pressure are likely more relevant than salinity in explaining these two species differences. A reduction in the fluidity of the cellular membrane will modify the diffusion of membrane molecules, including ions. Therefore, low temperatures and high pressures can affect fish osmoregulation in a complex manner^47^. Angiotensin II plays a relevant role in osmoregulation and affects blood pressure in fishes^47,50^. Thus, we hypothesize that ACE could help to modulate angiotensin II production, stabilize blood pressure under changing temperature conditions, reinforce homeostatic resilience and maintain a healthy state, which could contribute to increased longevity in *H. atlanticus*^64^.

**Fig. 6:**
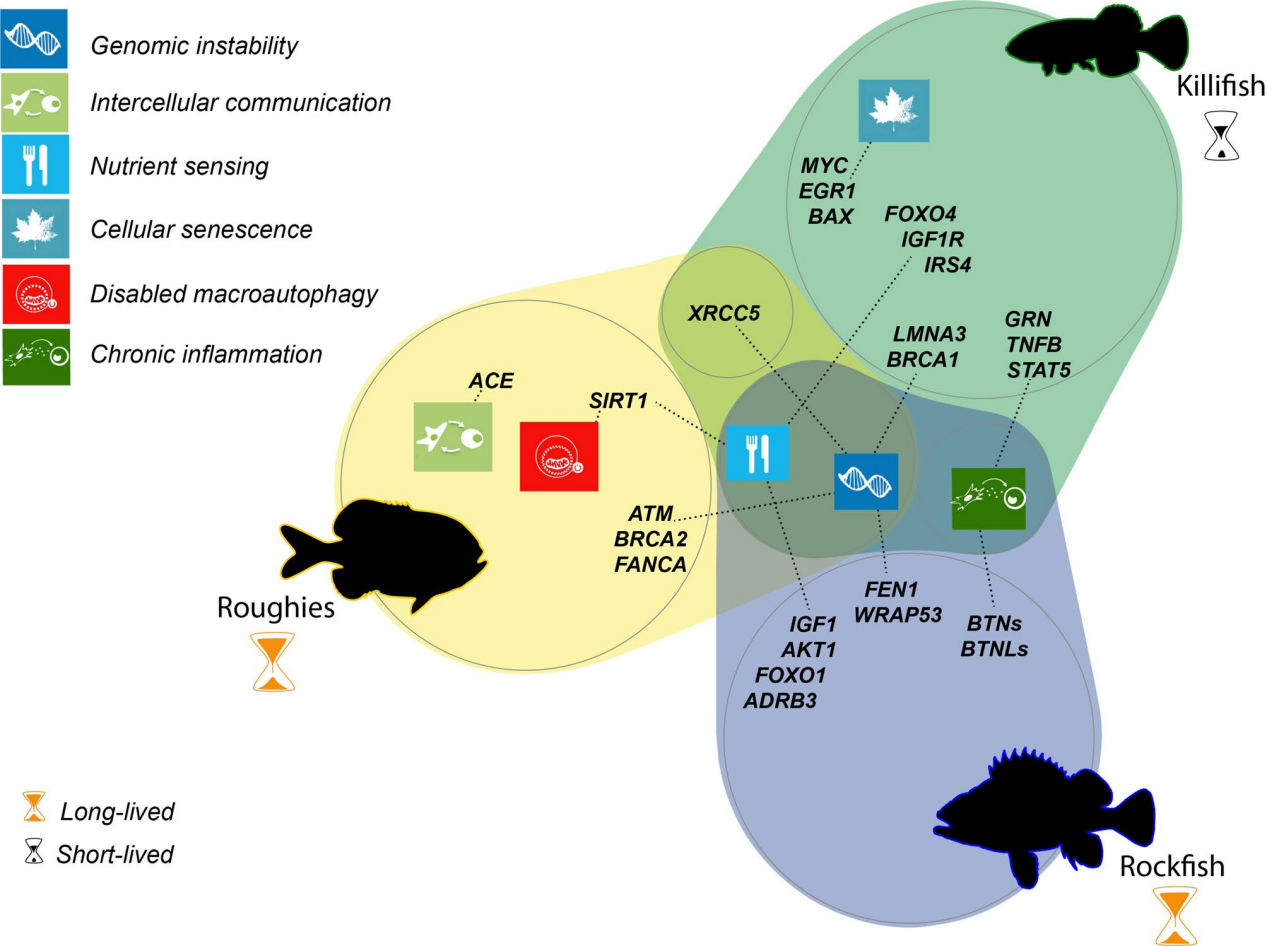
Hallmarks of aging and their highlighted genes relevant for longevity in killifish^16^, rockfish^3^ and roughies.

Food availability can be low at greater depths, and the area where *H. atlanticus* was collected (Challenger Plateau) is characterized by oligotrophic waters^65^. In contrast, primary production in the Cantabrian Sea, where *H. mediterraneus* is fished, is generally high^66^. These differences in habitat conditions could shape the adaptation of *H. atlanticus* to food limitations. As an epigenetic regulator, SIRT1 is also a relevant player in nutrient sensing via the mTOR pathway^36^, which can regulate nutrient input during a restricted diet and changes in metabolism. Accordingly, genes related to nutrient sensing were positively selected in both long-living killifish^16^ and rockfish^3^ (Fig. 6). Compared with *H. mediterraneus*, *H. atlanticus*, especially adults, has low metabolic rates^67,68^, which is evidenced by its larger size and later reproductive age. Although low metabolism is intuitively associated with dietary restriction, there is no evidence of an association between low food availability and low metabolic rates. This exponential decrease at greater depths could also be explained by the temperature changes and lack of visual predation, which relaxed the selective pressure for locomotory activity due to the lower number of predators^67,68^.

Deep-sea fishes are adapted to extreme environments characterized by high pressures and low light levels, temperatures, and food availability. At the same time, these environmental factors play a significant role in shaping the continuous increase in longevity with depth^69^. While this seems to be the general trend, there are also few exceptions, such as the hadal fish *Pseudoliparis swirei*^70^. In accordance with this trend and with the “adaptation hitch-hike model”^71^, we hypothesize that residue changes found in *H. atlanticus* could have been positively selected because they would entail adaptation to the deep-sea environment (namely, high pressures, low food availability and salinity changes), with increased longevity being an epiphenomenon of these adaptations. Nevertheless, further evolutionary analyses are needed to confirm this hypothesis.

## Conclusion

In this study, we present two de novo fish genomes, those of *H. mediterraneus* and *H. atlanticus*, and identify several point variants in relevant genes associated with the maintenance of genome stability, nutrient sensing, intercellular communication, and macroautophagy via manual annotation. Accordingly, we propose specific gene candidates that could contribute to the remarkable lifespan of *H. atlanticus* in comparison with *H. mediterraneus*. Nevertheless, further functional experiments are needed to assess the role of these variants in fish aging.

On the basis of our findings, we hypothesize that enhanced DNA repair, together with the modulation of intercellular communication, nutrient sensing, proteostasis, and macroautophagy pathways, could contribute to the adaptation of orange roughy to maintain cellular and organismal functions at great depths. At the same time, these adaptations would allow this species to live longer than *H. mediterraneus*. Overall, this work provides novel insights into the molecular mechanisms that confer an extraordinarily long lifespan on orange roughy.

## Methods

### Sample collection, processing and DNA/RNA isolation

No experiments were conducted in this study, and all methods were carried out in accordance with relevant guidelines and regulations. *Hoplostethus atlanticus* samples were collected via Thalassa Fisheries Support at the Challenger Plateau (quota management area ORH7A), west of New Zealand at a depth of 870 m in June 2020 (Fig. 1), under the commercial fishing permit of Talley’s Group Management Limited (Fisheries New Zealand Client Number 9760117). Immediately after collection, the samples were fixed with RNAlater at 4 °C overnight and stored at − 80 °C until they were sent to our laboratory. *H. mediterraneus* was kindly gifted by fishermen of the Avilés fish market, Asturias, Spain, in October 2019 after being stored at − 20 °C for 3 days. Afterward, we maintained frozen individuals at − 80 °C in our laboratory until extraction. Genomic DNA was isolated from muscular tissue via a standard phenol protocol. RNA was extracted from the muscle, gill and brain of *H. atlanticus* and from the muscle of *H. mediterraneus* via TRIzol reagent (Invitrogen). Prior to gDNA and RNA sequencing, 16S rDNA was amplified via polymerase chain reaction (PCR) (forward primer: 5′-CCGGTCTGAACTCAGATCACG-3′ and reverse primer: 5′-CGCCTGTTTAACAAAAACAT-3′) and checked for similarity in the NCBI database to confirm species identification.

### Genome sequencing, assembly and automatic annotation

The genomes of both *Hoplostethus* species were sequenced via a combination of PacBio Sequel 20 kb SMRTbell templates and Illumina TruSeq DNA PCR-free (350 bp insert) libraries. A consensus sequence was generated by assembling PacBio reads with the wtdbg2 (v2.3) assembler and mapping Illumina reads via Pilon (v1.21) to correct for base errors, misassemblies and gaps. The length of the resulting assembly was estimated by k-mer analysis, and its completeness was assessed with Benchmarking Universal Single-Copy Ortholog (BUSCO)^15^ analysis, using bacteria and eukaryote database. Protein-coding genes were automatically annotated using Maker (v2.31.8), and their functions were inferred on the basis of their homology to the protein sequence database (UniProt Swiss-Prot^72^) with Protein BLAST + (v2.7.1 +). The RNA was sequenced on the Illumina platform using Illumina TruSeq Stranded Total RNA (150 bp insert) libraries, and the reads were aligned to their respective genomes using STAR^73^. Genomic sequencing, assembly, automatic annotation and RNA sequencing were carried out by Macrogen commercial services (Seoul, Korea).

### Manual genome annotation

In parallel with this automatic analysis, we performed manually supervised annotation of the genomes of *H. mediterraneus* and *H. atlanticus* using BATI (Blast, Annotate, Tune, Iterate), a pipeline developed by our group^74^. Briefly, we curated a list of more than 400 genes selected a priori because of their involvement in aging, cancer, the stress response, telomere maintenance and DNA repair (Table 1). Each gene was selected on the basis of its role in age-related molecular processes^1,75^ and human conditions^76^, on the basis of the experience of our laboratory in these fields^2,4,56^, and following a detailed review of the available publications on each subject. Then, we used BATI to precisely define the intron/exon boundaries of the TBLASTN results. This procedure also helps identify novel homologues. In addition to each genome, the pipeline was fed reference protein sequences from *Danio rerio* and *S. salar* obtained from the NCBI database since they are phylogenetically closer to the targeted species, together with *Homo sapiens* proteins. In particularly difficult cases, we used the corresponding sequence from the automatic annotation of *H. mediterraneus* or *H. atlanticus* as a reference protein when available. All final gene sequences were compared to *H. sapiens* genes using the BLAST tool^77^ to validate the annotation (we considered only hits whose identity with *H. sapiens* homolog had an e value lower than 0.001).

### Genome comparison and candidate gene selection

After both annotations were complete, we performed multiple protein sequence alignments between *H. mediterraneus*, *H. atlanticus* and several other species (*H. sapiens, Pan troglodytes, Mus musculus*, *Heterocephalus glaber*, *Canis lupus familiaris*, *Gallus gallus*, *D. rerio*, *Oryzias latipes*, *Oncorhynchus mykiss*, *S. salar* and *N. furzeri*) for all the genes that presented only one copy in both *H. mediterraneus* and *H. atlanticus*. Within this list of single-copy genes, we looked for exclusive truncating variants, variants affecting known motifs according to the Conserved Domains database at the NCBI, UniProt and PubMed databases, and/or variants whose human counterparts are related to known genetic diseases according to the Clinvar database. We set a priori that a relevant point variant had to accomplish (1) being exclusive of one of the two fish species (or both) and absent in the rest of the species that we analysed and (2) being featured in at least one of the abovementioned databases. Additionally, relevant variants found exclusively in *H. atlanticus* were also annotated in other fishes with extreme lifespans: *S. aleutianus*, *A. fimbria*, *H. hippoglossus* and *A. anguilla* as long-lived fishes and *E. newberryi*, *P. occidentalis*, *Nothobranchius kuhntae* and *Hippocampus zosterae* as short-lived fishes. Alignments were performed using in-house software and sequences from the NCBI and Ensembl databases. The effect of each candidate variant in humans (as a proxy of the effect of the variant in *Hoplostethus*) was predicted via the PolyPhen^78^ and SIFT^79^ platforms. Moreover, in some cases, we performed a structural prediction of the specific residue change on the human protein model by using ChimeraX^80^. With respect to gene amplification analyses, genes with differential gene amplification among *Hoplostethus* species were further compared with the genomes of *D. rerio, O. latipes, O. mykiss, S. salar,* and *N. furzeri*. Only genes with unique gene amplification in one of the two *Hoplostethus* species were selected and subjected to a validation process.

### Validation

To validate the amplification of genes of interest, we performed PCRs with primer pairs that anneal to a target region with a differential variant in each copy (Supplementary Table S1). We tested the success of these reactions by performing electrophoresis of the resulting products in a 1.5% agarose gel. Genes whose amplification could not be validated by PCR or whose copy number differences disappeared because more copies were found in the other *Hoplostethus* species through PCR analysis were discarded. Additionally, point variants in residues with a relevant role in protein function were first validated with RNA-seq data from *H. mediterraneus* and *H. atlanticus*. Then, we performed PCRs aimed at analysing the affected nucleotide in each specific case. These products were sequenced via Sanger sequencing using an ABI PRISM 3130xl Genetic Analyser (Thermo Fisher).

### Significance Statement

Orange roughy (*H. atlanticus*) is one of the longest-lived fish species, with a lifespan of up to 250 years—a striking contrast to its congener, silver roughy (*Hoplostethus mediterraneus*), which lives for only 11 years. In this study, we present the de novo genomes of both orange and silver roughies and conduct comparative genomics analyses with more than 400 genes related to aging to uncover the genomic mechanisms that could underlie the marked differences in their lifespans. This research contributes to a deeper understanding of the molecular factors that drive longevity in fish.

### Supplementary Information

Supplementary Information.

## Data Availability

Assemblies have been deposited at the National Center for Biotechnology Information (NCBI, https://www.ncbi.nlm.nih.gov/), under Bioproject PRJNA1013967 and biosamples SAMN37314128 and SAMN37314129. RNAseq data have been deposited in Sequence Read Archive (SRA) under the same Bioproject number. Technical reports of both *Hoplostethus *species and MAKER2 predicted protein sequences can be downloaded from https://github.com/MariaPascualTorner/Hoplostethus.

## Code availability

The scripts for manual annotation (BATI) can be accessed at http://degradome.uniovi.es/downloads.html.
