# Supplementary material for: Insights into aging mechanisms from comparative genomics in orange and silver roughies
Source: Sci Rep. 2024 Aug 26;14:19748. doi: 10.1038/s41598-024-70642-w (PMC11347708; doi:10.1038/s41598-024-70642-w)

Insights into aging mechanisms from comparative genomics in orange and silver roughies

Dido Carrero[1,^], Maria Pascual-Torner[1,2,^,*], Diana Álvarez-Puente[1], Víctor Quesada[1], Claudia García-Gómez[1], Carlos López-Otín[1]

[1]Departamento de Bioquímica y Biología Molecular, Instituto Universitario de Oncología, Ciberonc, Universidad de Oviedo, Oviedo, Spain; [2]Observatorio Marino de Asturias, Departamento de Biología de Organismos y Sistemas, Universidad de Oviedo, Oviedo, Spain,

^*These authors contributed equally to this work*

***Send correspondence to**:*

Maria Pascual-Torner (mariapascual1.618@gmail.com)

Table S1. Specific oligonucleotides designed to PCR-validate point variants and frameshifts. *Hmed: H. mediterraneus, Hatl: H.atlanticus*

Gen	Species	Variant	Forward primer	Reverse primer
ACE	*Hatl*	R508H	GAGTGTGAAGTGAGGCGTTG	AGGTAGTTCAAGTGGACTGTCA
ATM	*Hmed*	R184Q	TGACATCACTCCACAGCAGT	TGGGGCAAGCGATTACAATC
ATM	*Hmed*	Q2177R	CGATCAACCCACTTTTGCCT	GTTGCAAGCTAGGTGTGGTAG
ATM	*Hatl*	L8F	CACACCTGGTTGATGGAAGC	CACAGGACAGAGACACAGGA
ATM	*Hatl*	V1085I	TGTGCTCCGTCGTAGTATCC	AGTGCTAAGTTTACGGGGCT
ATM	*Hatl*	I2914V	TCAAACCTGTGGCATTGTGA	TGATGGGTTGCAATGGTGTT
ATM	*Hatl*	V2937M	ATGAAGTGGTGGAGGCACAT	GGTGGAGTTGAGGGTAGCAT
BLM	*Hatl*	M827V	GGTGTGTTCCCGTGCATTAT	AATGCCACGGTCTGATTTGG
BLM	*Hatl*	I1039V	AGCTCTTTCTTGTGGCATGC	GGGTGGTCCTTACAGAAGCT
BRCA2	*Hmed*	S2807R	AGTTGATCATCCATGGGGCC	GCACAACGGAAAACAAAGCC
BRCA2	*Hatl*	S2807C	CATGCTTCACAAGGGTCGAC	ACAACGGAACACAAAGCCTC
CETP	*Hmed*	V29A	CCGATTGGGTGGAGGATAGG	TGACAGCCCTAGTTACGTTACA
DOCK8	*Hmed*	R398H	CTCCATTGTGTTCGCTCAGG	AGGGCACAGGAGGTTAGTTG
FANCA	*Hatl*	M427V	-	-
FANCI	*Hmed*	S979T	TGGGAATTGCACCGATACAG	CGACACAAACCACCCACTTT
IGF1R	*Hatl*	Frameshift	TGTGACTAAGTGCGCTCTCT	CATGCGAGCTATGTGAGGAC
IGF1R	*Hatl*	Frameshift	CTTCCTCTCACATCTGGATCCA	GGAGGCCGTCACCATAAGAG
IGF1R	*Hatl*	Frameshift	ACTACCAACTCTGACGTCTGG	CAGTCCTCCCTCCATCACAA
IL6	*Hatl*	Frameshift	TACTCACGCTCTTCTTCCGC	GCGCGGTTTGTTGCAGAT
NUDT1	*Hmed*	G58S	AAAATCCACTCTGTTGAACCTG	CCATGACGTCTGCATTGTCT
PROP1	*Hatl*	Frameshift	ACTGGGACTTCAACTGGGTC	GAGGGTGAGTGTGGAGACTG
SGK3	*Hmed*	I312V, T318M	TGAACAAAACCAACCAACACA	TCCACTGTCGGACTGTATGG
SIRT1	*Hatl*	V412I	TCTTCCTGCAGGTTGTTCCT	CGTACTTGGGATGAGAGCAA
SUV39H1	*Hatl*	Frameshift	AAGGTTGGTGAGGGTGTTGT	TCCCCTCTTCTTTATGCACTCA
XRCC5	*Hatl*	E417Q, M427T	CCAACCCTCAAGTAGGAGCA	ACCTGAGGGGGTGAACTTCT

Table S2. Results from RepeatMasker for *H. atlanticus* and *H. mediterraneus* genomes. SINEs (short interspersed transposable elements); LINEs (long interspersed nuclear element); LTR elements (long terminal repeats).

Species		hatl	hmed
**Retroelemen**	**SINEs**	0.12	0.1
	**Penelope**	0.01	0.01
	**LINEs**	2.12	1.68
	**LTR elements**	1.26	1.08
	**Total**	3.51	2.87
**DNA transposons**		2.77	2.55
**Rolling-circles**		0.07	0.04
**Unclassified**		0.03	0.02
**Total interspersed repeats**		6.3	5.43
**Small RNA**		0.06	0.04
**Satellites**		0.27	0.23
**Simple repeats**		4.15	4.9
**Low complexity repeats**		0.61	0.64
**Total**		17.77	13.85

Table S3. Manually annotated genes in *H. mediterraneus* and *H. atlanticus* with colors indicating their status. Blue identifies absent genes, green increased number of copies, beige unaltered, and red presence of stop codons or frame shift.

H.mediterraneus	*H. atlanticus*
APOL4	APOL4
APOL5	APOL5
C2	C2
COL6A3	COL6A3
F7	F7
A2M	A2M
ABCC4	ABCC4
ABCC8	ABCC8
ABO	ABO
ACD	ACD
ACE	ACE
ACTN3	ACTN3
ADA	ADA
ADAMTSL1	ADAMTSL1
ADARB1	ADARB1
ADARB2	ADARB2
ADCY5	ADCY5
ADIPOQ	ADIPOQ
AGER	AGER
AGPAT2	AGPAT2
AGT	AGT
AGTR1	AGTR1
AGTR2	AGTR2
AHSG	AHSG
AIF1	AIF1
AIFM1	AIFM1
AKT1	AKT1
AKT1S1	AKT1S1
AKT3	AKT3
ALDH2	ALDH2
APBB2	APBB2
APEX1	APEX1
APOA1	APOA1
APOA2	APOA2
APOA4	APOA4
APOA5	APOA5
APOB	APOB
APOC1	APOC1
APOC2	APOC2
APOC3	APOC3
APOC4	APOC4
APOD	APOD
APOE	APOE
APOF	APOF
APOH	APOH
APOL1	APOL1
APOL2	APOL2
APOL3	APOL3
APOL6	APOL6
APOM	APOM
APOO	APOO
APP	APP
ARHGAP1	ARHGAP1
ARNTL	ARNTL
ARSA	ARSA
ASIC2	ASIC2
ATG4C	ATG4C
ATM	ATM
ATP5O	ATP5O
ATR	ATR
ATRIP	ATRIP
ATXN2	ATXN2
BAG6	BAG6
BANF1	BANF1
BDNF	BDNF
BIN2	BIN2
BLM	BLM
BNIP2	BNIP2
BNIPL	BNIPL
BRCA1	BRCA1
BRCA2	BRCA2
BRE	BRE
BRIP1	BRIP1
BSCL2	BSCL2
BTBD19	BTBD19
BTG3	BTG3
BTNL2	BTNL2
BUB1B	BUB1B

BUB3	BUB3
C1QTNF5	C1QTNF5
C7ORF50	C7ORF50
CACNA1C	CACNA1C
CAMK4	CAMK4
CARS	CARS
CASP5	CASP5
CASP8	CASP8
CAT	CAT
CAV1	CAV1
CCL5	CCL5
CDC42	CDC42
CDH4	CDH4
CDK14	CDK14
CDK6	CDK6
CDK7	CDK7
CDKN1A	CDKN1A
CDKN1B	CDKN1B
CDKN2A	CDKN2A
CDKN2B	CDKN2B
CEBPA	CEBPA
CEBPB	CEBPB
CETP	CETP
CGNL1	CGNL1
CHEK1	CHEK1
CHEK2	CHEK2
CHIT1	CHIT1
CISD2	CISD2
CLOCK	CLOCK
CLSTN2	CLSTN2
CLU	CLU
CLYBL	CLYBL
COL5A1	COL5A1
COQ7	COQ7
CSF1R	CSF1R
CSNK1E	CSNK1E
CTC1	CTC1
CTF1	CTF1
CTNNA2	CTNNA2
CTNNA3	CTNNA3
CYC1	CYC1
CYP1B1	CYP1B1
DAGLA	DAGLA
DCLRE1A	DCLRE1A
DCLRE1B	DCLRE1B
DCLRE1C	DCLRE1C
DCPS	DCPS
DDB1	DDB1
DDB2	DDB2
DGAT1	DGAT1
DKC1	DKC1
DOCK8	DOCK8
DOT1L	DOT1L
DRD4	DRD4
EBF3	EBF3
EEF1A1	EEF1A1
EFEMP1	EFEMP1
EGFR	EGFR
EIF4E3	EIF4E3
EIF4EBP1	EIF4EBP1
EIF5A2	EIF5A2
ELN	ELN
ELOVL2	ELOVL2
ENOX1	ENOX1
ENOX2	ENOX2
ERCC1	ERCC1
ERCC2	ERCC2
ERCC3	ERCC3
ERCC4	ERCC4
ERCC5	ERCC5
ERCC6	ERCC6
ERCC8	ERCC8
ERGIC1	ERGIC1
ESR1	ESR1
ESRRG	ESRRG
EXO1	EXO1
FADS1	FADS1
FAM13A	FAM13A
FAM19A5	FAM19A5
FANCA	FANCA

FANCB	FANCB
FANCC	FANCC
FANCD2	FANCD2
FANCE	FANCE
FANCF	FANCF
FANCG	FANCG
FANCI	FANCI
FANCL	FANCL
FANCM	FANCM
FGFR1	FGFR1
FSHR	FSHR
GAPDH	GAPDH
GAR1	GAR1
GATA4	GATA4
GCLC	GCLC
GCLM	GCLM
GH1	GH1
GHRH	GHRH
GHRHR	GHRHR
GHRL	GHRL
GHSR	GHSR
GPX4	GPX4
GRAMD1B	GRAMD1B
GRN	GRN
GSK3A	GSK3A
GSK3B	GSK3B
GSR	GSR
GSTA1	GSTA1
GSTA2	GSTA2
GSTA3	GSTA3
GSTA4	GSTA4
GSTT1	GSTT1
GSTZ1	GSTZ1
GTF2H5	GTF2H5
H2AFX	H2AFX
H6PD	H6PD
HDAC1	HDAC1
HDAC2	HDAC2
HDAC3	HDAC3
HDAC4	HDAC4
HDAC5	HDAC5
HDAC6	HDAC6
HDAC7	HDAC7
HDAC8	HDAC8
HDAC9	HDAC9
HECW2	HECW2
HELLS	HELLS
HFE	HFE
HIF1A	HIF1A
HIP1	HIP1
HLA-DQB1	HLA-DQB1
HLA-DRB1	HLA-DRB1
HMOX1	HMOX1
HNRNPD	HNRNPD
HP	HP
HPCAL1	HPCAL1
HRAS	HRAS
HSPA9	HSPA9
HTRA2	HTRA2
ICAM1	ICAM1
IFNG	IFNG
IGF1R	IGF1R
IGF2R	IGF2R
IGFBP1	IGFBP1
IGFBP2	IGFBP2
IGFBP3	IGFBP3
IGFBP4	IGFBP4
IGFBP5	IGFBP5
IGFBP6	IGFBP6
IGFBP7	IGFBP7
IKBKB	IKBKB
IL10	IL10
IL12A	IL12A
IL12RB2	IL12RB2
IL18	IL18
IL6	IL6
IRS1	IRS1
IRS2	IRS2
ITGB3	ITGB3
JAK2	JAK2

KL	KL
LEP	LEP
LEPR	LEPR
LIG1	LIG1
LIG3	LIG3
LIG4	LIG4
LINGO1	LINGO1
LINGO2	LINGO2
LMNA	LMNA
LMNB1	LMNB1
LMNB2	LMNB2
LMO4	LMO4
LPA	LPA
LRP4	LRP4
LY6G6F	LY6G6F
LY86	LY86
MAPK3	MAPK3
MAPK9	MAPK9
METTL1	METTL1
MGMT	MGMT
MIF	MIF
MLH1	MLH1
MLH3	MLH3
MRE11A	MRE11A
MSH2	MSH2
MSH3	MSH3
MSH4	MSH4
MSH5	MSH5
MSH6	MSH6
MSRA	MSRA
MSTN	MSTN
MT1A	MT1A
MT1E	MT1E
MT2A	MT2A
MTOR	MTOR
MTTP	MTTP
MYT1L	MYT1L
NAV2	NAV2
NBN	NBN
NCAM2	NCAM2
NCOR2	NCOR2
NEIL1	NEIL1
NEIL2	NEIL2
NETO1	NETO1
NFE2L1	NFE2L1
NFE2L2	NFE2L2
NFKB1	NFKB1
NFKBIA	NFKBIA
NHEJ1	NHEJ1
NLN	NLN
NLRC5	NLRC5
NLRP3	NLRP3
NPAT	NPAT
NR3C1	NR3C1
NUDT1	NUDT1
OGDH	OGDH
PALB2	PALB2
PARP2	PARP2
PARVG	PARVG
PAX4	PAX4

PCK1	PCK1
PCMT1	PCMT1
PCNA	PCNA
PGPEP1	PGPEP1
PICALM	PICALM
PIK3CA	PIK3CA
PLAT	PLAT
PLAU	PLAU
PLXNA4	PLXNA4
POLA1	POLA1
POLA2	POLA2
POLB	POLB
POLD1	POLD1
POLD2	POLD2
POLD3	POLD3
POLD4	POLD4
POLE	POLE
POLE2	POLE2
POLE3	POLE3
POLE4	POLE4
POLG	POLG
POLG2	POLG2
POLH	POLH
POLI	POLI
POLK	POLK
POLL	POLL
POLM	POLM
POLN	POLN
POLQ	POLQ
PON1	PON1
POT1	POT1
POU5F1	POU5F1
PPARG	PPARG
PPARGC1A	PPARGC1A
PPM1D	PPM1D
PRKCA	PRKCA
PRKDC	PRKDC
PRKN	PRKN
PROP1	PROP1
PRR5	PRR5
PRUNE2	PRUNE2
PSEN1	PSEN1
PSEN2	PSEN2
PTEN	PTEN
PTH	PTH
RAE1	RAE1
RECQL	RECQL
RECQL4	RECQL4
RECQL5	RECQL5
RHOA	RHOA
RICTOR	RICTOR
RMI1	RMI1
RMI2	RMI2
RPA1	RPA1
RPA2	RPA2
RPTOR	RPTOR
RTEL1	RTEL1
RYR3	RYR3
SDC4	SDC4
SDHC	SDHC

SERPINE1	SERPINE1
SGK1	SGK1
SGK2	SGK2
SGK3	SGK3
SH2B3	SH2B3
SIRT1	SIRT1
SIRT2	SIRT2
SIRT3	SIRT3
SIRT4	SIRT4
SIRT5	SIRT5
SIRT6	SIRT6
SIRT7	SIRT7
SLC9A3R2	SLC9A3R2
SLX4	SLX4
SNCA	SNCA
SOCS1	SOCS1
SOCS2	SOCS2
SOCS3	SOCS3
SORCS1	SORCS1
SORCS2	SORCS2
SPRTN	SPRTN
SQSTM1	SQSTM1
ST3GAL3	ST3GAL3
STK24	STK24
STUB1	STUB1
SUCLA2	SUCLA2
SUV39H1	SUV39H1
SVEP1	SVEP1
TENM4	TENM4
TERF1	TERF1
TERF2	TERF2
TERT	TERT
TGFA	TGFA
TGFB1	TGFB1
TH	TH
TMEM151B	TMEM151B
TMEM2	TMEM2
TNF	TNF
TOMM40	TOMM40
TOP3B	TOP3B
TOX	TOX
TP53	TP53
TP63	TP63
TP73	TP73
TPP1	TPP1
TPP2	TPP2
TSFM	TSFM
TSHR	TSHR
TXN	TXN
UCP1	UCP1
UCP2	UCP2
UCP3	UCP3
VASH1	VASH1
VWA5A	VWA5A
WRN	WRN
XDH	XDH
XPA	XPA
XPC	XPC
XRCC1	XRCC1
XRCC2	XRCC2
XRCC3	XRCC3
XRCC4	XRCC4
XRCC5	XRCC5
XRCC6	XRCC6
YBX2	YBX2
YTHDF2	YTHDF2
YWHAG	YWHAG
ZMPSTE24	ZMPSTE24

Table S4. Gene variants and amplifications discarded after PCR validation

Gene	Species	Variant/Copy number	Observation
ATM	*Hmed*	A2451E	Variant discarded through Sanger sequencing
BRCA2	*Hmed*	E2850A	Variant discarded through Sanger sequencing
HTRA2	*Hmed*	Stop codon	Stop codon discarded through Sanger sequencing
NHEJ1	*Hatl*	Copy number	4 (out of 7) copies were to similar to be able to be amplified using unique primers, therefore can not be validated. *H. mediterraneus* had 3 copies
TMEM151B	*Hatl*	Frame shift	Frameshifts discarded through Sanger sequencing

Table S5. List of genes with copy number variations between *H. mediterraneus* and *H. atlanticus*, that were discarded after comparison with other fish species, and the number of copies found in each species. Numbers in parenthesis represent number of copies that were transcriptionally active and bold numbers indicate copy number modified after manual annotation**.**

	H. mediterraneus	*H. atlanticus*	*D. rerio*	*O. latipes*	*O. mykiss*	*S. salar*	*N. furzeri*
APBB2	2	3	**1**	**4**	**9**	**7**	**6**
APOB	3	5	**3**	3	5	2	3
APOH	3	2	**0**	2	**3**	**3**	1
APOL3	4	6	**6**	2	3	4	1
CDKN2B	0	1	**1**	**1**	1	**1**	1
CYC1	2	3	**1**	**0**	2	**4**	1
F7	5	4	**1**	2	5	5	3
GSK3B	2	4	**3**	**1**	4	**4**	1
IGFR1	3	2	**2**	2	4	4	2
IL6	1	0	**0**	**1**	**0**	**0**	**0**
LINGO2	3	2	2	**2**	3	6	2
LRP4	3 (1)	1 (1)	**1**	**1**	**2**	**2**	**1**
NHEJ1	3 (1)	7 (4)	1	1	2	2	**1**
PRKN	1	2	1	**1**	2	6	1
SDC4	2	1	1	**2**	2	4	2
CASP8	3	2	**3**	2	**6**	7	**2**

Table S6. List of genes of butyrophillin family each copy number in *H. mediterraneus* and *H. atlanticus*, and other fish species compared

Gene	*H. mediterraneus*	*H. atlanticus*	*D. rerio*	*O. latipes*	*O. mykiss*	*S. salar*	*N. furzeri*
BTN1A1	10	7	6	13	58	36	6
BTN2A1	2	2	4	2	48	8	1
BTN2A2	2	1	5	0	1	13	1
BTN3A1	1	3	0	1	1	6	0
BTN3A2	1	1	0	0	0	0	0
BTN3A3	6	8	4	0	1	8	6
BTNL2	2	2	18	0	1	4	1
BTNL3	0	0	5	0	1	3	0
BTNL8	0	4	0	0	4	2	0
BTNL9	0	0	0	0	0	0	0

Table S7. List of genes of flavin-containing monooxygenase family with each copy number in *H. mediterraneus* and *H. atlanticus*, and other fish species

Gene	*H. mediterraneus*	*H. atlanticus*	*D. rerio*	*O. latipes*	*O. mykiss*	*S. salar*	*N. furzeri*
FMO1	0	0	0	0	0	0	0
FMO2	0	2	1	1	1	1	2
FMO3	0	0	0	0	0	0	0
FMO4	0	0	0	1	0	0	0
FMO5	4	3	2	3	4	4	5

Table S8. List of paralogs for each candidate gene discussed in the manuscript, along with the number of orthologs present in *H. mediterraneus* and *H. atlanticus*

**Candidate gene**	**Gene orthologs**	***H.mediterraneus***	***H.atlanticus***
ACE	*ACE*	1	1
	ACE2	2	2
ATM	*ATM*	1	1
	ATR	1	1
	MTOR	1	1
	SMG1	1	1
	PRKDC	1	1
	TRRAP	1	1
BLM	*BLM*	1	1
	WRN	0	0
	RECQL	1	1
	RECQL4	1	1
	RECQL5	1	1
CETP	*CETP*	1	1
	BPI	1	1
	BPIFA1	0	0
	BPIFA2	0	0
	BPIFA3	0	0
	BPIFB1	0	0
	BPIFB2	0	0
	BPIFB3	0	0
	BPIFB4	0	0
	BPIFB6	0	0
	BPIFC	1	0
	LBP	0	0
	PLTP	1	1
DOCK8	*DOCK8*	1	1
	DOCK1	1	1
	DOCK2	1	1
	DOCK3	2	2
	DOCK4	1	1
	DOCK5	1	1
	DOCK6	1	1
	DOCK7	1	1
	DOCK9	2	2
	DOCK10	1	1
	DOCK11	1	1
NUDT1	*NUDT1*	1	1
	NUDT12	1	1
	NUDT17	1	1
	NUDT18	1	1
SIRT1	*SIRT1*	1	1
	SIRT2	1	1
	SIRT3	2	2
	SIRT4	1	1
	SIRT5	1	1
	SIRT6	1	1
	SIRT7	1	1
XRCC5	*XRCC5*	1	1
	XRCC1	1	1
	XRCC2	1	1
	XRCC3	1	1
	XRCC4	2	2
	XRCC6	1	1
BRCA2, FANCA, FANCI	*BRCA1*	1	1
	BRCA2	1	1
	BRIP1	1	1
	ERCC4	1	1
	FANCA	1	1
	FANCB	1	1
	FANCC	1	1
	FANCD2	1	1
	FANCE	1	1
	FANCF	1	1
	FANCG	1	1
	FANCI	1	1
	FANCL	1	1
	FANCM	1	1
	PALB2	1	1
	SLX4	1	1

Figure S1. Partial amino acid sequence alignment of BRCA2 and phylogenetic three indicating the specific amino acid change in a wider number of species. p.S2807 variant is highlighted with a blue box and an arrow in the alignment.

BRCA2

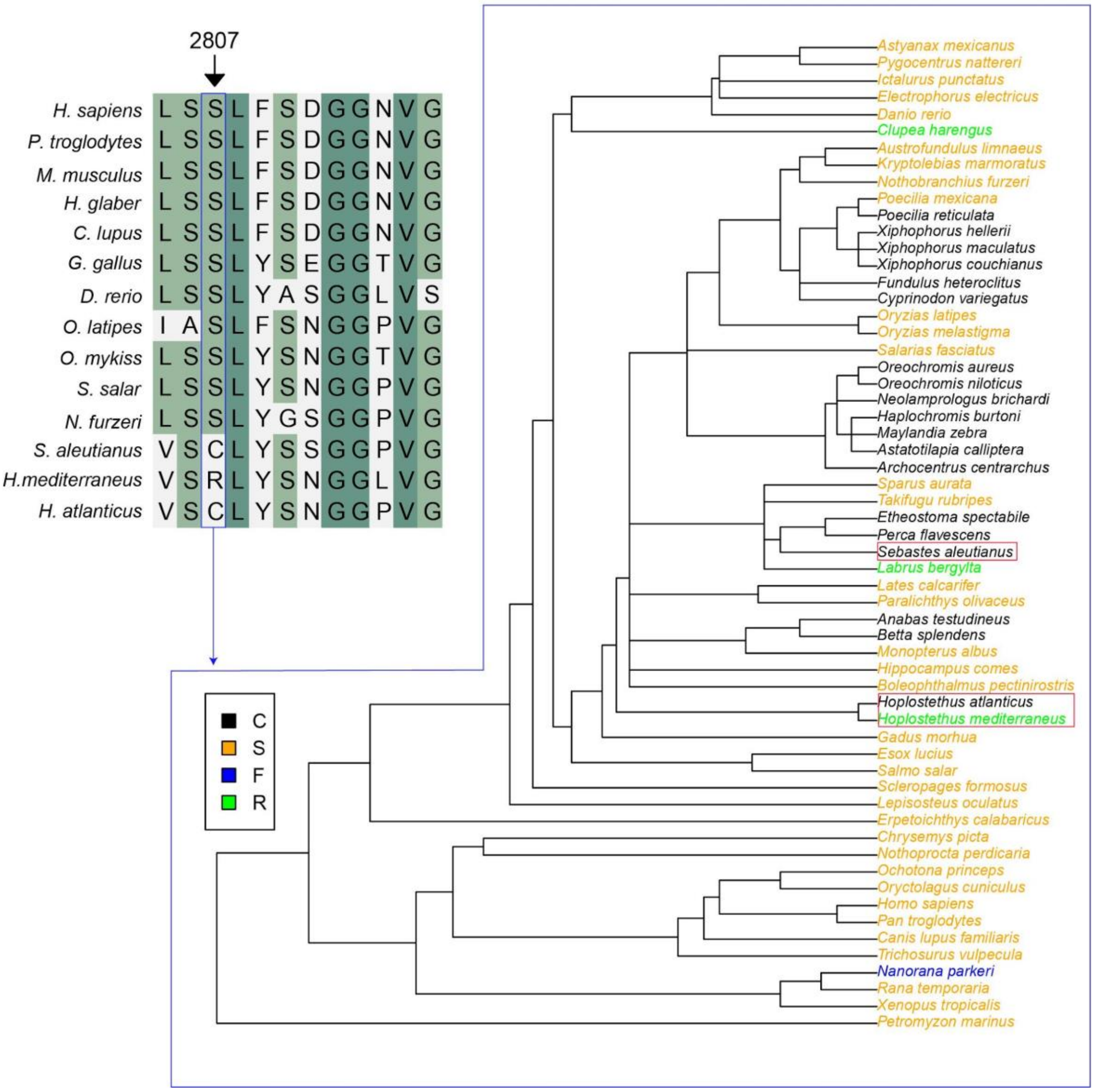

Figure S2. Plot of temperature and salinity profiles in Challenger Plateau area. The plot was built with '*ggplot2* ' in R platform, using datasets from 3 points (Float 5904438 - Cycle 125, Float 5903301 - Cycle 170, Float 5904814 - Cycle 64) in Argo float data (https://argo.ucsd.edu/). Shade area indicates the range of depths of *H. atlanticus*

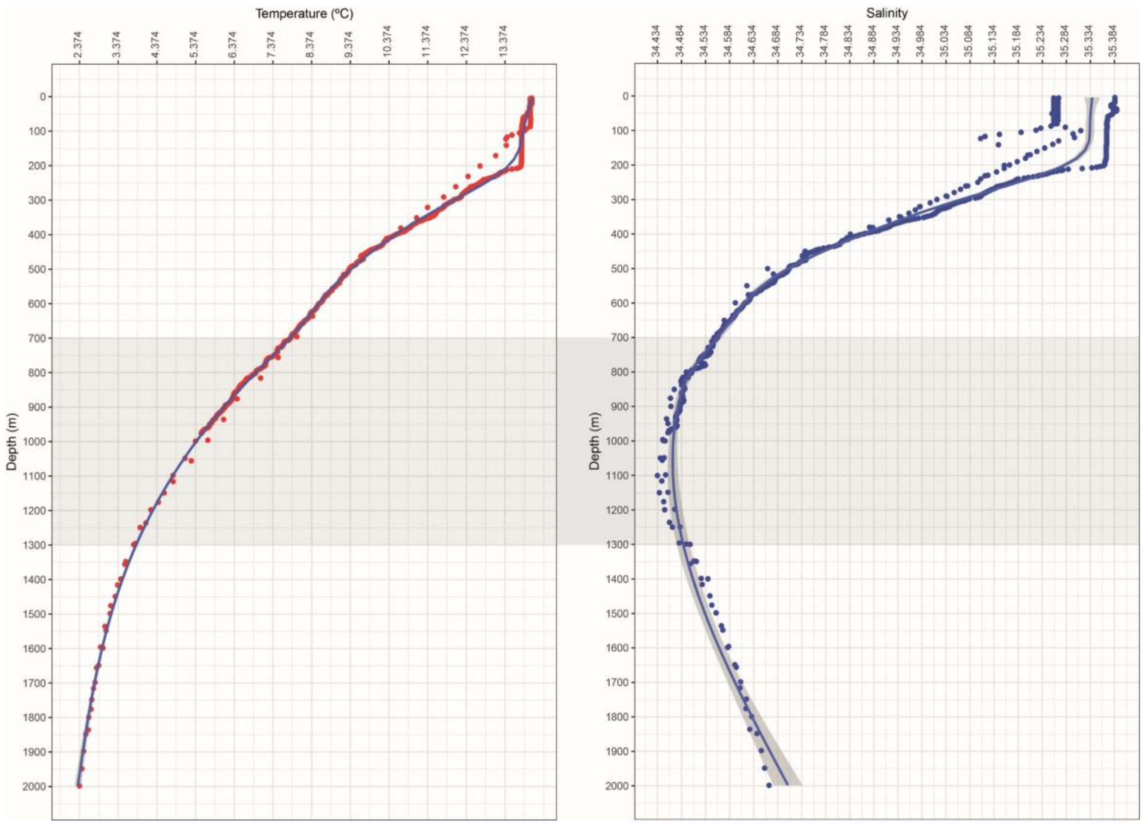

Supplement: Supplementary file 1 — Supplementary Information. [file 41598_2024_70642_MOESM1_ESM.pdf]
